# Comparative *in vitro *study of the antimicrobial activities of different commercial antibiotic products of vancomycin

**DOI:** 10.1186/1472-6904-11-9

**Published:** 2011-07-21

**Authors:** Jorge A Diaz, Edelberto Silva, Maria J Arias, María Garzón

**Affiliations:** 1 Universidad Nacional de Colombia, Facultad de Ciencias, Departamento de Farmacia, Laboratorio de Asesorías e Investigaciones en Microbiología, 472. Ciudad Universitaria. Carrera 30 Calle 45. A.A.14490. Bogotá D. C. Colombia; 2 Vitalis Pharmaceutical, Proyectos Especiales, Carrera 7 No 156-80. Oficina No 1104. Bogotá D. C. Colombia

## Abstract

**Background:**

One of the most critical problems about antimicrobial therapy is the increasing resistance to antibiotics. Previous studies have shown that there is a direct relation between erroneous prescription, dosage, route, duration of the therapy and the antibiotics resistance. Other important point is the uncertainty about the quality of the prescribed medicines. Some physicians believe that generic drugs are not as effective as innovator ones, so it is very important to have evidence that shows that all commercialized drugs are suitable for therapeutic use.

**Methods:**

Microbial assays were used to establish the potency, the Minimal Inhibitory Concentrations (MICs), the Minimal Bactericidal Concentration (MBCs), the critical concentrations, and the production of spontaneous mutants that are resistant to vancomycin.

**Results:**

The microbial assay was validated in order to determine the Vancomycin potency of the tasted samples. All the products showed that have potency values between 90 - 115% (USP requirement). The products behave similarly because the MICs, The MBCs, the critical concentrations, the critical concentrations ratios between standard and samples, and the production of spontaneous mutants don't have significant differences.

**Conclusions:**

All products analyzed by microbiological tests, show that both trademarks and generics do not have statistical variability and the answer of antimicrobial activity Show also that they are pharmaceutical equivalents.

## Background

Pharmaceutical products, especially antibiotics, must comply with standards of quality, efficacy and reliability, attributes that are determined by various authorities [[1,2], and [3]]. A discussion about the quality and efficacy of generic antibiotics has taken place in recent decades. This discussion has included presentations in congress and research articles in which the authors have shown that some products do not meet regulatory standards [4,5] and that their behavior is not similar in animal models [6,7]

Some antibiotics must be analyzed using biological assays (e.g., penicillin, amikacyn, vancomycin, and neomycin) [2]. These products are measured by their potency or biological activity compared against an international standard. Therefore, the commercial products must be similar in composition to the international reference standard [7]. With antibiotics like vancomycin, if the commercial products do not fulfill the requirements of pharmacopeia, their behavior and performance could put a patient's health in danger.

Biological assays and other analytical procedures must be validated before they are applied in the analysis of the content of the antibiotic under study because, otherwise, neither the information or data generated nor conclusions obtained will be reliable [3]. Our worry arises from the fact that some researchers confuse a "gold standard" with an international reference standard for quantification. A gold standard is something that is a defined commercial product used as reference of performance in comparative studies. It is not a reference standard, but another commercial product with its own variation. Gold standards are established for purposes of bioequivalence and bioavailability studies [2], but in the case of IV antibiotics, the bioavailability is 100%, and therefore, pharmacodynamic studies must be supported with validated analytical results [2].

Our group has been focusing on developing validated techniques using proper international reference standards to evaluate the content or potency of commercial antibiotics. These techniques can be used in performance studies like those for the determination of a Minimal Inhibitory Concentration (MIC), Minimal Lethal Concentration, Critical Concentration and production of Spontaneous Mutants [8,9].

This paper presents the results for the evaluation of commercial products of vancomycin to describe some issues that are important in the evaluation of antibiotics.

## Methods

### Microorganisms

THE UNITED STATES PHARMACOPOEIA XXVII states that spores of *Bacillus subtilis *ATCC 6633 are the source of this microorganism used to develop a microbiological assay for evaluating the potencies of vancomycin products. For MIC and MBC studies, we used *Acinetobacter baumanii *strains 59, 139, 147 and 173, *Enterococcus gallinarum, Streptococcus faecalis *ATC 29212, a nosocomial strain 319623 and a vancomycin-sensitive strain, *Escherichia coli *strains 39, 50 and 69, *Klebsiella pneumoniae *strains 1, 43, 63, 65 and 207, *Pseudomonas aeruginosa *strains 42, 74, 151, 157, and HE1, *Staphylococcus aureus *strains 287, 291 and ATCC 25923, and *Morganella morganii *HE2. All of the microorganisms were grown in Mueller Hinton (MH) broth (incubated at 35°C for 24 h). Each strain was then plated on MH agar to obtain isolated colonies, which were then used to make larger cultures in MH medium. The cultures were harvested with cryopreservation broth. A portion of each was kept in a cryovial at -70°C, and the other portion was used to prepare a suspension with 25% transmittance at 600 nm (25%T) to develop *in vitro *assays. These suspensions were kept in cryovials at - 70°C.

### Analytical Bioassay

An analytical bioassay was established and validated for vancomycin. First, the proper concentration range was determined, and then the linearity, precision, specificity and stability of the compound in question were assessed [2,3]. All of the samples were evaluated with this analytical bioassay under the chosen conditions.

### Minimal Inhibitory Concentration (MIC) and Minimal Bactericidal Concentration (MBC)

Assays to assess these parameters were developed in two parts. (1) **Preparation of inocula: **the number of colony forming units (CFUs) was determined for each suspension at 25%T to prepare inocula of 1-5 × 10^6 ^CFUs/ml. (2) **MIC and MBC determination by micro-dilution: **samples were diluted to 2 mg/ml for evaluation. Using a multichannel pipette, 100 μl Mueller Hinton Broth was placed in each well of a 96-well ELISA plate, with 200 μl in column 12. Next, 100 μl of the antibiotic solution (2 mg/ml) was placed in the first column and thoroughly mixed by pipetting. From these wells, 100 μl was added to the second column and mixed, and this procedure was repeated up to column 10, after which the 100-μl portion was discarded. Columns 11 and 12 were positive and negative controls, respectively. Each row (A to H) represented a different sample to be analyzed. Each inoculum (100 μl) was then pipetted into each microplate, which was incubated at 37°C for 24 h. Growth in the wells was assessed. The lowest dilution showing no growth, the first dilution with growth, and the two controls were plated onto MH agar. The **MIC **was defined as the lowest dilution that showed no growth on the ELISA plate but showed growth on MH agar. The **MBC **was defined as the lowest dilution that did not show growth on either the ELISA plate or MH agar [10].

### Critical Concentration (CC)

The CC was determined similarly to the analytical bioassay. The inocula for **MIC **and **MBC **determinations and two-fold serial dilutions of each sample from 993 to 31,03 μg/ml were used (The batch of Vancomycin USP standard has a potency of 99300 μg per vial). The halo of inhibition was measured, and the crown length (Χ) was calculated (the inhibition halo diameter minus the reservoir diameter divided by 2). The log concentration vs. Χ^2 ^was plotted, and a linear regression (*y *= *mx *+ *b*) was applied. The y-intercept (*b*) is equivalent to the log of the CC [10].

### Spontaneous mutants

Spontaneous mutation was analyzed similarly to the analytical bioassay. Again, the inocula for the **MIC **and **MBC **determinations were used. Specific microorganisms and dilutions were selected after determinations of critical concentrations. On each plate, a dilution of the USP standard and samples of the same concentration were used.

### Samples

Commercial products purchased from the pharmacies of different hospitals in Bogotá, D. C. Colombia, were analyzed. They included trademarked products and generic products of vancomycin. All of the samples had declared contents of 500mg. They were all diluted in sterile water in 100 ml volumetric flasks. The solutions were divided into 5-ml fractions for storage at -70°C and were diluted to **1 **mg/ml to develop the analytical bioassays.

### Statistical Analysis

All the assays were performed three times, and the statistical tool of Microsoft Excel^® ^was applied to analyze the dates.

## Results

### Analytical Bioassay

**Figure 1:**
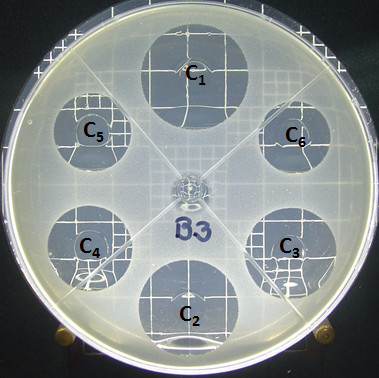
**Bioassay of Vancomycin (USP standard) against *Bacillus subtilis *ATCC 6633**.

The United Stated Pharmacopoeia XXVII recommends *Bacillus subtilis *ATCC 6633 as the biological organism to use to develop the analytical bioassay for vancomycin products. Figure 1 shows the results of this bioassay.

#### Determination of concentration range, incubation time and culture medium pH

**Figure 2:**
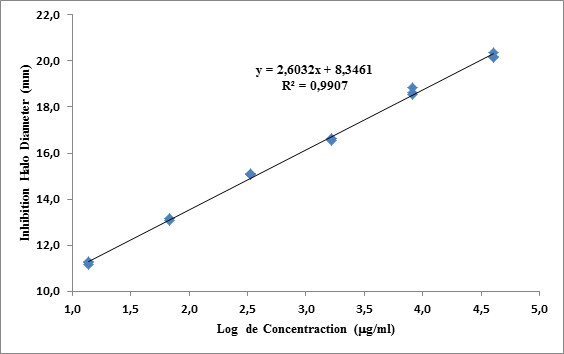
**Calibration curve of Vancomycin (USP standard) used to evaluate the linearity of the optimal concentration range**.

Ten concentrations were used to determine the concentration range (two-fold dilutions from 1005 to 1.96 μg/ml, because this batch of Vancomycin USP standard has a potency of 100500 μg/vial). Table 1 shows that the best linearity was in the range between C3 and C8 (251.25 to 7.85 μg/ml) (R^2 ^= 0.9907, Figure 2).

**Table 1:** Evaluation of the range of concentrations for Vancomycin (USP standard)

Concentration Range		Equation		
**From**	**To**	**Slope**	**Intercept**	**R ^2^**

C1	C6	2.462680435	6.415123505	0.996221756
C2	C7	2.324635129	7.259328533	0.989384179
**C3**	**C8**	**2.270224777**	**7.362386326**	**0.990682625**
C4	C9	2.367915749	6.864768679	0.98750859

Cited on page 6

The assay required an 8 to 10 h incubation time at 37°C. This incubation is shorter than many common assays, which require between 18 and 24 h.

The results for Vancomycin show that a pH of 6.4 or 6.5 is optimal because growth was abundant and homogenous, and inhibition haloes were well defined at this pH (Table 2).

**Table 2:** Evaluation of the pH effect on linearity

pH	Equation	R^2^	Incubation Time
5.4	y = 1.8463x + 14.882	0.9806	8 hours
5.9	y = 2.3895x + 19.128	0.9898	8 hours
**6.4**	**y = 1.5517x + 11.817**	**0.9977**	**8 **hours
**6.5**	**y = 2.134x + 7.4113**	**0.9975**	**8 **hours
7	y = 1.7824 + 11.212	0.9794	9 hours
7.5	y = 1.875x + 11.009	0.9663	10 hours
8	y = 2.3651x + 9.3311	0.9763	11 hours

Cited on page 6

#### Linearity

In Tables 3 and 4, the concentration of antibiotic correlates well with the diameter of the zone of inhibition.

**Table 3:** Evaluation of the linearity of Vancomycin

Test	HYPOTHESIS	Experimental t	Theoretical t	Decision
**Slope**	H_0_: m = 0 H_1_: m ≠0	19.7	2.120	Reject H_0_
**Intercept**	H_0_: b = 0 H_1_: b ≠0	125.3	2.120	Reject H_0_
**Correlation**	H_0_: R = 0 H_1_: R ≠ 0	67.5	2.120	Reject H_0_

Cited on page 6

**Table 4:** Regression analysis by analysis of variance (ANOVA).

Test	HYPOTHESIS	Experimental t	Theoretical t	Decision
**Regression**	H_0_: There is no regression H_1_: There is regression	146.6	4.670	Reject H_0_
**Deviation from Linearity**	H_0_: There is no deviation from linearity H_1_: There is a deviation from linearity	-3.0	3.71	Accept H_0_

Cited on page 6

From this point on, the selected concentrations will be designated C1 to C6 for clarity.

#### Precision

The reproducibility and between-day precision of our assays were evaluated in several ways. Reproducibility was studied by determining the coefficient of variation, which was less than 1% and was acceptable for analytical assays in the pharmaceutical industry (Table 5).

**Table 5:** Reproducibility of assays using Vancomycin (Cochran Test)

Concentration (mg/ml)	251.25	165.63	62.85	31.41	15.70	7.85
**Standard deviation**	0.096	0.076	0.237	0.084	0.100	0.270
**Variance Coefficient (%)**	0.4759	0.408422	1.4576	0.559623	0.7061	2.2425
**Variance (S^2^)**	0.0092	0.00583	0.0113	0.00707	0.0099	0.01213

**Sum (S^2^)**						0.05544

Cited on page 6

The between-day precision was also analyzed. Analysis of variance (ANOVA) showed that, for the antibiotic evaluated, the results of assays performed on different days did not significantly differ (Table 6).

**Table 6:** ANOVA of the between-day precision of assays using Vancomycin

Concentration	Experimental F	Theoretical F	Decision
**C1**	0.041	4.96	Accept H_0_
**C2**	0.047	4.96	Accept H_0_
**C3**	0.069	4.96	Accept H_0_
**C4**	0.093	4.96	Accept H_0_
**C5**	0.128	4.96	Accept H_0_
**C6**	0.182	4.96	Accept H_0_

Cited on page 6

#### Stability

The stability of each compound during the experimental period was verified. Solutions of vancomycin in water and phosphate buffer, pH 4.5 (1005 μg/ml; USP Standard), were incubated at 37°C, 18°C and 4°C, and samples were taken after 24, **48**, and 86 hours or seven and fifteen days of incubation. The samples (Vancomycin Standard Solution) under different treatments, were diluted fromC1 to C6 to perform the relation Log Concentration vs. Halo Diameter Inhibition, and the results were plotted and compared to reveal any reduction in antibiotic activity (i.e., a decrease in the diameter of the zone of inhibition).

From the equation *y *= *mx *+ *b*, where *y *represents the inhibition zone diameter and *x *represents the log of the concentration, changes in the value of *b *indicate changes in activity. If there is no change in the intercept, the antibiotic is stable. If the value of *b *decreases, this trend indicates instability or a loss of activity.

The solutions showed a slight decrease in the intercept values after 24 h of at each storage temperature (Tables 7 and 8). From this result, it appears that the molecule remained stable during our assays (48 hours at 37°C). Therefore, the assay results reflect the exact potency of the product.

**Table 7:** Stability of Vancomycin in water for injection at 4°C, 18°C and 37°C

Time	4°C			18°C			37°C		
	
	Slope	Intercept	R^2^	Slope	Intercept	R^2^	Slope	Intercept	R^2^
0 h	1.5177	12.556	0.9913	1.5177	12.556	0.9913	1.5177	12.556	0.9913
24 h	1.5305	12.543	0.9908	1.5241	12.518	0.9916	1.5063	12.5210	0.9900
48 h	1.522	12.544	0.9919	1.518	12.501	0.9926	1.4936	12.495	0.9924
86 h	1.5224	12.509	0.9916	1.5178	12.461	0.9924	1.4981	12.4720	0.9928
7 days	1.5165	12.4780	0.9919	1.5217	12.342	0.9935	1.4742	12.3040	0.9904
15 days	1.5247	12.3460	0.9921	1.5041	12.273	0.9923	1.4425	12.1980	0.9916

Cited on page 7

**Table 8:** Stability of Vancomycin in phosphate buffer, pH 4

Time	4°C			18°C			37°C		
	
	Slope	Intercept	R^2^	Slope	Intercept	R^2^	Slope	Intercept	R^2^
0 h	1.4807	12.799	0.9916	1.4807	12.799	0.9916	1.4807	12.799	0.9916
24 h	1.4910	12.7640	0.9917	1.4833	12.764	0.9917	1.5195	12.5150	0.9916
48 h	1.487	12.733	0.9924	1.4747	12.716	0.9928	1.509	12.497	0.9922
86 h	1.4787	12.7260	0.9933	1.4701	12.689	0.9927	1.5057	12.4700	0.9915
7 days	1.4804	12.6510	0.9925	1.4766	12.571	0.9931	1.4966	12.3800	0.9922
15 days	1.4826	12.5170	0.9937	1.4566	12.505	0.9932	1.4887	12.2510	0.9926

Cited on page 7

#### Specificity

To test specificity, solutions of the antibiotics were incubated at 50°C. The vancomycin solutions lost a small amount of activity (3% to 4%) after 15 days, but after 30 days, there was no longer any activity, meaning that vancomycin was the only molecule in solution responsible for the antimicrobial activity (Table 9).

**Table 9:** Stability of Vancomycin in phosphate buffer, pH 4

Time	Phosphate Buffer, pH 4.5			Water For Injection		
	
	Slope	Intercept	R^2^	Slope	Intercept	R^2^
0 h	1.4807	12.799	0.9916	1.5177	12.556	0.9913
24 h	1.5268	12.4310	0.9909	1.5059	12.4640	0.9905
48 h	1.4924	12.479	0.9912	1.4907	12.409	0.993
86 h	1.4894	12.4530	0.9914	1.4939	12.3520	0.9930
7 days	1.4515	12.3970	0.9869	1.4569	12.3230	0.9897
15 days	1.4226	12.3060	0.9855	1.4343	12.2370	0.9907
30 days	NDA			NDA		

ND: Non detectable activity

Cited on page 7

### Sample analysis

The samples were analyzed with the previously validated assay. The results were quantified using the statistical method described by Hewitt (1977). Table 10 shows the content of vancomycin in the samples purchased, and in each case, the values fulfill the criteria laid out by USP XXV II for intravenous vancomycin: **"...Contents no less than 90% and no more than 115% of Vancomycin, calculated on anhydrous base of the quantity registered of Vancomycin"**.

**Table 10:** Potency of the commercial samples of vancomycin

Samples	Potency
1	
2	0.995
3	1.012
4	1.005
5	1.100
6	0.936
7	1.124
8	1.032
9	
10	1.064
11	
12	1.019
13	1.023
14	1.150
15	1.108
16	0.9859
17	1.107
18	1.047
19	
20	0.981
21	1.019
22	1.011
23	
24	1.003
25	1.023
26	1.011
27	0.961
28	
29	1.062
30	

Cited on pages 7 and 9

### Minimal inhibitory and bactericidal concentrations

**Figure 3:**
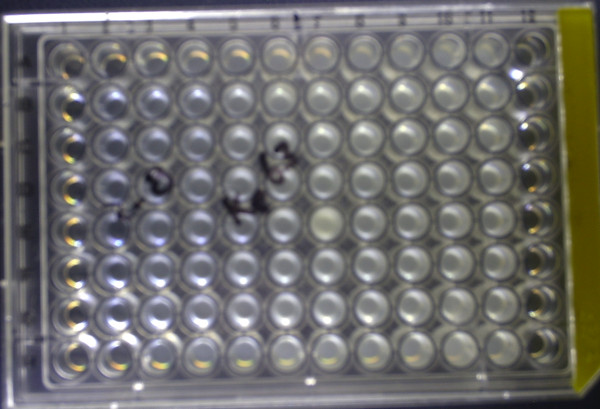
**MIC assays of vancomycin products against *K. pneumoniae *63**.

Using the previously described methods, the samples were analyzed in groups of seven per plate, and each plate was inoculated with a single bacterial strain. The first row of the plate contained the USP standard; the other seven rows contained the samples. Figure 3 shows the results for vancomycin products. The plates showed the same performance for the standard as for the samples.

Growth was inhibited at the same concentration of each sample. After transfer onto MH agar, there was no growth in concentrations C1 to C5 or C12, but there was growth in C6 to C11. This result means that the antibiotic has an MBC but no MIC. The MBC is C5 for the USP standard and for all the samples. For all of the samples, using all of the microorganisms evaluated, the results showed that the samples had the same performances at each repetition of the assay (Table 11 includes results for only some samples as an illustration).

**Table 11:** Determination of MICs and MBCs for Vancomycin (USP standard)

Microorganism	MIC (μg/ml)			MBC (μg/ml)		
	
	Std	M1	M2	Std	M1	M2
*A. baumanii *59	62.06	62.06	62.06	124.13	124.13	124.13
*A. baumanii *139	124.13	124.13	124.13	248.25	248.25	248.25
*A. baumanii *147	993	993	993	ND	ND	ND
*A. baumanii *173	62.06	62.06	62.06	124.13	124.13	124.13
*E. faecalis*	1.93	1.93	1.93	3.88	3.88	3.88
*E. faecalis *ATCC 29212	7.76	7.76	7.76	15.52	15.52	15.52
*E. faecalis *319623	62.06	62.06	62.06	124.13	124.13	124.13
*E. gallinarum*	ND	ND	ND	124.13	124.13	124.13
*E. coli *39	124.13	124.13	124.13	248.25	248.25	248.25
*E. coli *50	124.13	124.13	124.13	248.25	248.25	248.25
*E. coli *69	496.50	496.50	496.50	993.00	993.00	993.00
*K. pneumoniae *1	ND	ND	ND	496.50	496.50	496.50
*K. pneumoniae *43	496.5	496.5	496.5	993.00	993.00	993.00
*K. pneumoniae *63	993.00	993.00	993.00	ND	ND	ND
*K. pneumoniae *65	993.00	993.00	993.00	ND	ND	ND
*K. pneumoniae *207	496.00	496.00	496.00	993.00	993.00	993.00
*Ps. aeruginosa *42	1.94	1.94	1.94	3.88	3.88	3.88
*Ps. aeruginosa *74	1.94	1.94	1.94	3.88	3.88	3.88
*Ps. aeruginosa *151	993.00	993.00	993.00	ND	ND	ND
*Ps. aeruginosa *157	993.00	993.00	993.00	ND	ND	ND
*Ps. aeruginosa *HE1	993.00	993.00	993.00	ND	ND	ND
*St. Aureus *287	1.94	1.94	1.94	3.88	3.88	3.88
*St. Aureus *291	1.94	1.94	1.94	3.88	3.88	3.88
*St. Aureus *ATCC 25923	1.94	1.94	1.94	3.88	3.88	3.88
*M. morganii *HE2	496.50	496.50	496.50	993.00	993.00	993.00

Cited on pages 7 and 9

### Critical concentration (CC)

The CC is the minimum concentration that inhibits microorganism growth. It occurs at the limit of the inhibition halo. It is a measure of a microorganism's sensitivity and can be different from the MIC, which is determined under different conditions. The CC can be defined mathematically as Ln(CC) = Ln(C_O_) - X^2^/DT_O_, where CC is the critical concentration, C_O _is the antibiotic concentration in the reservoir, X is the length of the crown (see above), D is the diffusion coefficient, and T_O _is the critical time. The intercept of a plot of Ln (C_O_) vs. X^2 ^is the Ln of CC [7].

**Figure 4:**
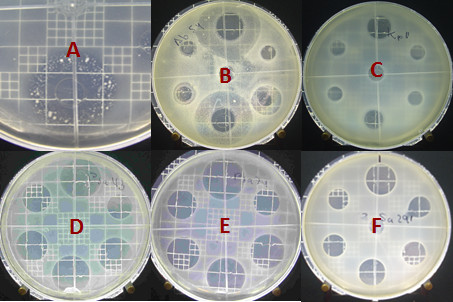
**Zones of inhibition produced by Vancomycin against (A) *E. gallinarum*, (B) *A. baumanii *54, (C) *K. pneumoniae *1, (D) *P. aeruginosa *43, (E) *P. aeruginosa *74 and (F) *S. aureus *291**.

**Figure 5:**
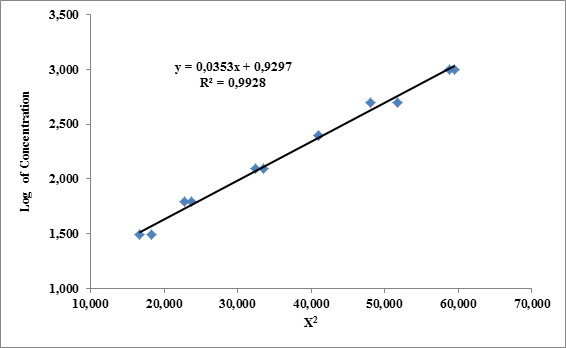
**Determination of critical concentration of Vancomycin against *P. aeruginosa *74**.

Figure 4 shows the different behaviors of the microorganisms tested with the vancomycin standard. In Figures 4A and 4B, the microorganisms exhibited growth of spontaneous mutants. Figure 4C shows a microorganism resistant to vancomycin, and, finally, Figures 4D, E and 4F correspond to microorganisms with well-defined haloes, allowing for a comparison of the performances of the products tested for development. A well-defined inhibition halo was the selection criterion for evaluating CCs. For the CC assays, *E. faecalis*, *E. faecalis *ATCC 29212, *E. faecalis *319623, *A. baumanii *59, *E. gallinarum*, *P. aeruginosa *43 and 74, *S. aureus *281, 291 and ATCC 25923 were selected. Figure 5 shows the correlation of X^2 ^with the log of antibiotic concentration. The regression equation is *y*= 0.0353*x *+ 0.9297, and *b *is therefore 0.9287. The CC is equivalent to antilog (0.9297), i.e., 8.506 μg/ml.

The CC values for the different vancomycin products showed no significant differences, meaning that the products behaved in similar ways against the different microorganisms tested (Table 12). On this basis, the generic products meet all of the quality standards applied to the pharmaceutical products and perform as well as the newest versions of these products.

**Table 12:** Critical concentrations (μg/ml) of different samples of Vancomycin against various microorganisms.

Sample	***E. f***.	*E. f*. 29212	*E. f*. 319623	*A. b*. 59	***E. g***.	*P. a*. 43	*P. a*. 74	*S. a*. 281	*S. a*. 291	*S. a*. 25923
Standard	13.251	14.098	26.733	7.712	14.725	10.932	8.586	9.951	12.473	13.108
M2	13.332	14.173	26.826	7.735	14.850	10.988	8.646	10.044	12.558	13.164
M3	13.050	14.170	26.505	7.759	14.993	10.977	8.745	10.032	12.764	13.410
M4	13.166	14.041	26.630	7.670	15.076	10.870	8.635	9.991	12.682	13.202
M5	12.961	14.566	26.160	8.305	14.798	11.716	9.474	10.941	13.753	14.662
M6	14.495	13.338	28.017	7.355	13.974	10.308	8.016	9.280	11.725	12.324
M7	12.523	14.237	26.540	7.856	16.029	12.348	9.666	11.143	14.076	14.729
M8	13.441	13.627	27.530	8.053	15.008	11.248	8.903	10.138	13.220	13.440
M13	13.052	14.193	26.574	8.003	14.955	11.146	8.908	10.306	12.713	13.489
M14	13.792	15.904	28.612	8.481	15.291	12.621	9.897	11.480	14.454	15.286
M15	13.791	15.673	27.431	8.190	15.700	11.969	9.500	11.015	13.820	14.539
M16	13.483	14.326	26.535	7.698	14.991	10.743	8.458	9.795	12.363	12.986
M17	13.720	15.128	27.261	8.068	15.794	12.163	9.519	11.006	13.786	14.510
M18	13.697	14.790	26.915	7.720	15.196	11.671	8.965	10.384	13.427	13.807
M20	13.568	14.405	27.117	7.660	14.535	10.760	8.506	9.835	12.249	12.886
M21	13.192	14.632	26.985	7.857	16.048	11.178	8.759	10.091	12.625	13.413
M22	14.067	13.946	26.536	7.774	15.571	11.309	8.741	10.057	12.769	13.437
M24	13.334	14.046	26.701	7.856	14.655	10.895	8.639	10.016	12.418	13.125
M26	13.882	14.592	26.519	7.638	15.409	11.116	8.739	10.038	12.671	13.299
M27	12.741	13.544	25.775	7.474	14.126	10.499	8.326	9.612	12.200	12.595
M29	13.571	14.008	26.770	8.274	15.281	11.105	9.143	10.404	12.998	13.887

Cited on pages 8 and 10

In addition, the ratio between the sample CCs and standard CCs are similar to the ratios of antibiotic contents. In other words, all samples perform the same with regard to their antimicrobial activities *in vitro *(Table 13).

**Table 13:** Ratios of sample CC/standard CC for Vancomycin

SAMPLE	MICROORGANISMS										Ratio Median	Potency
			
	***E. f***.	*E. f*. 29212	*E. f*. 319623	*A. b*. 59	***E. g***.	*P. a*. 43	*P. a*. 74	*S. a*. 281	*S. a*. 291	*S. a*. 25923		
Standard												
M2	1.006	1.005	1.003	1.003	1.008	1.005	1.007	1.009	1.007	1.004	1.006	0.995
M3	0.985	1.005	0.991	1.006	1.018	1.004	1.018	1.008	1.023	1.023	1.008	1.012
M4	0.994	0.996	0.996	0.995	1.024	0.994	1.006	1.004	1.017	1.007	1.003	1.005
M5	0.978	1.033	0.979	1.077	1.005	1.072	1.103	1.100	1.103	1.119	1.057	1.100
M6	1.094	0.946	1.048	0.954	0.949	0.943	0.934	0.933	0.940	0.940	0.968	0.936
M7	0.945	1.010	0.993	1.019	1.089	1.130	1.126	1.120	1.128	1.124	1.068	1.124
M8	1.014	0.967	1.030	1.044	1.019	1.029	1.037	1.019	1.060	1.025	1.024	1.032
M13	0.985	1.007	0.994	1.038	1.016	1.020	1.038	1.036	1.019	1.029	1.018	1.023
M14	1.041	1.128	1.070	1.100	1.038	1.155	1.153	1.154	1.159	1.166	1.116	1.150
M15	1.041	1.112	1.026	1.062	1.066	1.095	1.106	1.107	1.108	1.109	1.083	1.108
M16	1.018	1.016	0.993	0.998	1.018	0.983	0.985	0.984	0.991	0.991	0.998	0.986
M17	1.035	1.073	1.020	1.046	1.073	1.113	1.109	1.106	1.105	1.107	1.079	1.107
M18	1.034	1.049	1.007	1.001	1.032	1.068	1.044	1.044	1.076	1.053	1.041	1.047
M20	1.024	1.022	1.014	0.993	0.987	0.984	0.991	0.988	0.982	0.983	0.997	0.981
M21	0.996	1.038	1.009	1.019	1.090	1.023	1.020	1.014	1.012	1.023	1.024	1.019
M22	1.062	0.989	0.993	1.008	1.057	1.035	1.018	1.011	1.024	1.025	1.022	1.011
M24	1.006	0.996	0.999	1.019	0.995	0.997	1.006	1.006	0.996	1.001	1.002	1.003
M26	1.048	1.035	0.992	0.990	1.046	1.017	1.018	1.009	1.016	1.015	1.019	1.011
M27	0.962	0.961	0.964	0.969	0.959	0.960	0.970	0.966	0.978	0.961	0.965	0.961
M29	1.024	0.994	1.001	1.073	1.038	1.016	1.065	1.046	1.042	1.059	1.036	1.062

### Spontaneous mutants

It was noted in the previous assays that some strains produced spontaneous mutants (Figure 4A), as indicated by the appearance of colonies within the inhibition halo. Therefore, an assay to assess spontaneous mutation was developed with appropriate concentrations of antibiotics. Each experimental setup included an agar plate inoculated with a test strain. Of the six reservoirs, two contained standard solutions and the other four contained sample solutions. The numbers of mutants produced by the standard and sample solutions were counted after incubation.

**Figure 6:**
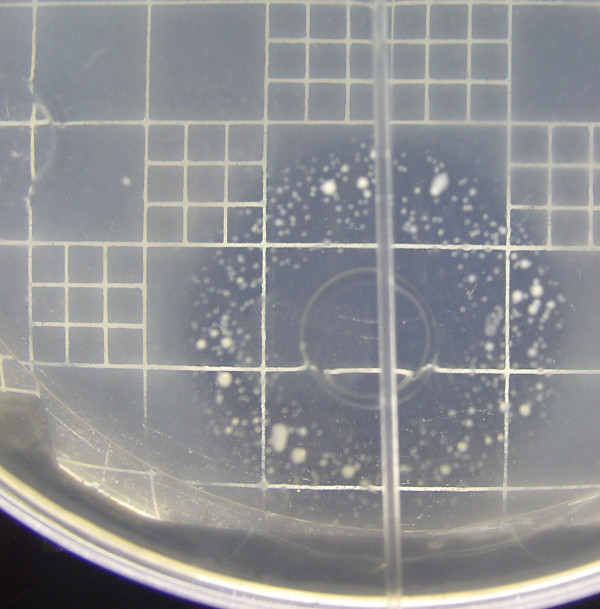
**Production of spontaneous vancomycin-resistant mutants of *E. gallinarum***.

For the spontaneous mutant assays, the strains selected were S. *aureus *291 as a control strain (showing no production of spontaneous mutants) and *A. baumanii *54 and *E. gallinarum *as mutant producing strains. After statistical analysis, the results (Table 14) showed no significant differences between the products in the production of spontaneous mutants for any of the strains tested (Figure 6).

**Table 14:** Spontaneous mutant production in the diffusion gel assay for vancomycin products

Sample	Mutants of *A. baumanii *54		Mutants of *E. gallinarum*	
	
	Median	σ	Median	σ
Standard	106.17	1.47	96.500	5.089
M2	111.00	1.00	100.667	2.082
M3	104.33	1.53	98.333	1.528
M4	106.67	2.08	104.667	5.686
M5	103.67	0.58	99.000	2.000
M6	109.00	1.00	96.000	2.000
M7	110.67	1.53	100.667	1.155
M8	108.67	1.53	98.667	1.155
M10	104.00	1.73	95.667	1.528
M12	110.67	1.53	93.333	2.517
M13	106.33	1.53	94.000	3.000
M14	106.67	2.52	101.000	1.000
M15	110.67	1.15	99.333	1.155
M17	105.33	1.15	93.667	3.786
M18	104.33	2.08	96.000	1.000
M20	109.33	1.53	100.667	0.577
M22	112.00	1.00	103.000	3.000
M24	105.33	1.53	96.000	1.000
M26	109.33	1.53	100.667	0.577
M27	112.00	1.00	103.000	3.000
M29	105.33	1.53	96.000	1.000

F	10.026		4.424	
Prob.	0.001		0.005	
VCF	1.706		1.706	

Cited on page 9

## Discussion

Despite the fact that USP Pharmacopoeia assesses the bioassay conditions for vancomycin evaluation, the bioassay was validated following the suggestions of the specialized literature [1-3], to assure the certainty of results concerning the sample contents. The experiment to evaluate assay performance showed that it fulfilled the assay requirements (linearity, repeatability, precision). In the assay, the best linearity was shown over the range of 251.25 μg/ml to 7.85 μg/ml, i.e., the correlation was the highest (R^2 ^= 0.9907). The reproducibility and between-day precision of both assays had coefficients of variation less than 1%, and ANOVA showed no significant differences at any concentration. Antibiotic activity remained stable over the course of the assay at the selected temperature. Finally, the inhibition assay results were due only to the molecules evaluated. In conclusion, the assay was exact and accurate with reproducible results.

Our results were generally similar to those of Zuluaga et al. (2009), but with some differences. Zuluaga et al. (2009) proposed a comparison of the performances of all samples by linear correlation against the performance of the original compound to determine pharmaceutical equivalence. This approach is problematic because the commercial products exhibit some differences in their potency. The USP Pharmacopoeia XXVII states **"...Contents no less than 90% and no more than 115% of Vancomycin, calculated on anhydrous base of the quantity registered of Vancomycin"**, are acceptable. Therefore, if we use a reference element for which there is uncertainty about its content, a sample could be assessed against different potencies. For example, if the commercial sample has 90% of the potency of Vancomycin, the potency of the sample under study will be overvalued, but if the reference sample has 115% of the potency, the sample under study will be undervalued. Finally, we strongly recommend that an antibiotic must be evaluated against an international reference standard by established and validated bioassays using an appropriate test microorganism and conditions. Then, the conclusions about the samples contents will be certain.

Analyses of commercial versions of the antibiotics tested (brand-name and generic products) indicate that all of the samples can be considered pharmaceutical equivalents because they all fulfill the standards of the USP Pharmacopoeia (Table 10). In the study by Zuluaga et al. (2009), the performance of all samples was similar to the innovator, and the results were accurate and reproducible, which means that all of the producers of this antibiotic are using similar parameters to manufacture their products.

The MIC and MBC results obtained with different pathogenic strains showed no differences between samples (Tables10 and 11), which is probably because the samples were pharmaceutical equivalents. We conclude that generic and novel products perform equally well. In other words, the generic products evaluated in this study fulfill the requirements to be considered for use in antimicrobial therapy.

We also designed an assay to determine critical concentrations using a few selected strains to confirm that all of the generic products evaluated were effective in antimicrobial therapy. The results showed no significant differences among samples (Table 12). Moreover, the ratios between the CC of the standard and those of the different samples were similar to their potency levels (Tables 13).

Along the same lines, an assay was designed to determine the production of spontaneous mutants in diffusion gel assays. The results again showed that all the samples behaved similarly, leading us to conclude that none of the samples studied markedly differ in their antimicrobial activities. That is, generic and brand name products that comply with the international specifications for manufacturing pharmaceutical products behave similarly to novel products.

Our results are different from those of other studies [5,6]. Those studies were conducted using the newest product as a "standard of comparison," but the researchers did not take into account that a commercial product may have a range of content between 90% and 120%. Consequently, there would be great variability in the results with respect to the performance of the antibiotic. For instance, if the novel drug product has a hypothetical content of 120% relative to the declared content on the label, and the generic product has a hypothetical content of 90%, then the effective content of the generic product would be 75% (90/120) of the novel drug. This scenario could produce misleading results because although both products fulfill the content requirements, the first is at the upper limit and the second at the lower limit.

It has been proposed that generic antibiotics behave differently from innovator products against pathogenic microorganisms [5,6]. This is possible if the generic antibiotic does not fulfill the quality standards for that pharmaceutical product (e.g., purity or content). For instance, contaminants in generic drugs could interfere with their antibiotic activities.

Vesga et al (2009) reported that none of the vancomycin products have differences in *in vitro *assays; they had no differences in potency, MIC or MBC. Also, in time-kill curves and single-dose serum Pharmacokinetics (PK) in infected mouse there were no differences. However, the pharmacodynamic study had very odd results; the products tested did not behave like the innovator *in vitro*. We think that these results should be reanalyzed or retested because at the lower concentration, the generics have a better antimicrobial activity than the innovator, but in the higher concentrations, these behaviors change. The free antibiotic in the serum is the only chemical responsible for the antimicrobial activity and they showed in the PK model that all of the antibiotics diffuse into the blood in an equivalent way; so, they should behave against the same microorganism in an equivalent way.

## Conclusions

All of the samples analyzed by standardized, microbiological methods fulfill the requirements for content according to USP XXVII. They all show the same antimicrobial behavior because they have similar MIC, MBC and CC values and produce similar numbers of mutants.

## Abbreviations

MIC: Minimal Inhibitory Concentration; MBC: Minimal Bactericidal Concentration; CC: Critical Concentration; C1: Concentration 1; C2: Concentration 2; C10: Concentration 10; *A. b.: Acinetobacter baumanii; S. f.: Streptococcus faecalis; E. g.: Enterococcus gallinarum; E. c.: Escherichia coli; K. p.: Klebsiella pneumonia; P. a.: Pseudomonas aeruginosa; S. a.: Staphylococcus aureus*; M1: Sample 1; M2: Sample 2, ...

## Competing interests

Diaz and Silva received financial support for lectures from Vitalis S. A. to participate in national scientific meetings in Colombia. The present study was a joint venture between the Science Faculty of National University of Colombia and Vitalis Pharmaceutical. And was also financed by Vitalis Pharmaceutical.

## Authors' contributions

MG, a student at the National University of Colombia, jointly developed a process to validate the quantitative assay for vancomycin for their theses in Pharmaceutical Chemistry. MJA was the project administrator and contributed to article redaction. JAD and ES conceived the study, obtained necessary funding, designed and directed the execution and analysis of data, edited the manuscript and approved it for publication.

All the authors read and are in agreement with the whole all of article text.

## Pre-publication history

The pre-publication history for this paper can be accessed here:

http://www.biomedcentral.com/1472-6904/11/9/prepub
